# Use of a model to understand the synergies underlying the antibacterial mechanism of H_2_O_2_-producing honeys

**DOI:** 10.1038/s41598-020-74937-6

**Published:** 2020-10-19

**Authors:** Maria Masoura, Paolo Passaretti, Tim W. Overton, Pete A. Lund, Konstantinos Gkatzionis

**Affiliations:** 1 School of Chemical Engineering, University of Birmingham, Birmingham, B15 2SA UK; 2 Institute of Microbiology and Infection (IMI), University of Birmingham, Birmingham, B15 2SA UK; 3 Department of Food Science and Nutrition, School of the Environment, University of the Aegean, Lemnos, Greece

**Keywords:** Antimicrobials, Applied microbiology

## Abstract

Honey has been valued as a powerful antimicrobial since ancient times. However, the understanding of the underlying antibacterial mechanism is incomplete. The complexity and variability of honey composition represent a challenge to this scope. In this study, a simple model system was used to investigate the antibacterial effect of, and possible synergies between, the three main stressors present in honey: sugars, gluconic acid, and hydrogen peroxide (H_2_O_2_), which result from the enzymatic conversion of glucose on honey dilution. Our results demonstrated that the synergy of H_2_O_2_ and gluconic acid is essential for the antibacterial activity of honey. This synergy caused membrane depolarization, destruction of the cell wall, and eventually growth inhibition of *E. coli* K-12. The presence of H_2_O_2_ stimulated the generation of other long-lived ROS in a dose-dependent manner. Sugars caused osmosis-related morphological changes, however, decreased the toxicity of the H_2_O_2_/gluconic acid. The susceptibility of catalase and general stress response sigma factor mutants confirmed the synergy of the three stressors, which is enhanced at higher H_2_O_2_ concentrations. By monitoring cellular phenotypic changes caused by model honey, we explained how this can be bactericidal even though the antimicrobial compounds which it contains are at non-inhibitory concentrations.

## Introduction

The emergence of antibiotic resistance, one of the biggest worldwide public health concerns, has renewed interest in natural antimicrobials such as honey. Over recent decades, extensive research on honey has provided evidence of its medicinal properties^1^. This has allowed the clinical use of numerous products manufactured from medical-grade honey produced under standardized conditions^2^. These honeys originate from various floral sources and vary in antibacterial activity; therefore, the antibacterial strength does not appear to be limited to particular honey species. Wildflower, buckwheat, heather, and honeydew honeys are among the most antibacterial species^3,4^. To date, the greatest medical potential of honey is its application as a topical agent to wounds and skin infections because of its anti-inflammatory and antioxidant properties and broad-range antimicrobial activity^1^. However, a full understanding of the underlying antimicrobial mechanism of honey is required in order to take advantage of its full potential as a medicinal product.

Low osmolality (0.5–0.6 aw) was initially believed to be the main antimicrobial factor in honey. Later it was reported that diluted honeys have increased antibacterial strength due to the production of hydrogen peroxide (H_2_O_2_). This is caused by the oxidation of glucose into gluconic acid and H_2_O_2_ that occurs upon dilution^5^. The concentration of accumulated H_2_O_2_ ranges between 0.04 and 4 mM and for most honeys the maximum yield is achieved at dilution factors of 30–50%^6–11^. Gluconic acid, accumulating to a concentration of between 8.6 and 60 mM, is the most abundant acid in honey and the major determinant of its acidity (pH 3.4—4.5)^12^. However, the role of gluconic acid in the antimicrobial activity of honey has not been thoroughly investigated yet.

Recent studies reported that additional components of honey, other than H_2_O_2_, account for some of its antimicrobial activity^13^. This is partly explained by the fact that the accumulated H_2_O_2_ in honey is 900-fold lower than this used in medical disinfectants. The antimicrobial peptide bee defensin-1 has been shown to be bactericidal (at 0.5 μg/ml) by creating pores within the cell membrane of Gram-negative and -positive bacteria (i.e. *E.coli*, *S. enterica*, *S. aureus*)^14^. Besides that, the amount of def-1 found in honeys cannot be correlated with the total antibacterial activity^11^. Upon dilution, polyphenols found in honey either directly produce H_2_O_2_ or reduce Fe (III) to Fe (II) that further stimulate the production of H_2_O_2_ and other reactive oxygen species (ROS) via the Fenton reaction^11,15^. These reactions have been shown to cause lipid peroxidation and damage to bacterial cell proteins and DNA^4,16–18^. Also, MGO, generating during the natural degradation of the phytochemical dihydroxyacetone, was seen to cause oxidative stress by reacting with cellular proteins and the DNA. The presence of defensins and H_2_O_2_ enhanced the activity of MGO against a broad spectrum of bacteria^13^. However, since honeys of different floral and geographical origins differ greatly in composition, the contribution of each factor to the antimicrobial action remains obscure.

The studies quantifying the antibacterial effect of honeys (i.e. growth-based antimicrobial assays) outnumber those explaining the mechanism itself. However, the latter have shown that honey targets a series of events related to growth initiation, cell division and cell wall synthesis, all crucial for bacterial viability^4,10,15,19–22^. Microscopy has provided evidence of honey-induced phenotypic alterations on pathogenic bacteria (i.e. *P. aeruginosa* ATCC 10145 and *S. pyogenes* ATCC 19615), such as changes from coccoidal to rod-shaped cells and eventually inhibition of septation and cell division^23,24^. Flow cytometry (FC) has shown that honey increases the permeability of the outer membrane of Gram-negative bacteria (i.e. *E. coli* O157:H7, *P. aeruginosa*, *P. syringae*, and *S. enterica* serovar Typhimurium) by destroying the lipopolysaccharide layer and also changes the membrane potential in both Gram-negative (*E. coli*) and -positive (*S. aureus*) bacteria^25–27^.

Although H_2_O_2_ highlighted as a major antimicrobial agent in honey, recent studies reported that other honey components, such as phytochemicals, may enhance or inhibit the activity of the former^28^. However, sugars, gluconic acid and H_2_O_2_, as co-exist during the glucose oxidation, represent the most abundant stressors. Osmotic, acid, and oxidative stresses have been studied individually and by focusing on quantifying their effects rather than understanding of the mechanism underlying the synergy between them. The complexity of honey composition represents a big challenge to study the antibacterial mechanism induced by the three main stressors. Thus, a model system that combines sugars, gluconic acid, and H_2_O_2_, in a range of concentrations as were quantified on honey dilution, is expected to give a better understanding of their synergies. Although the presence of other antimicrobial components in honey (MGO, phytochemicals, def-1) were seen to affect its antibacterial activity, here it was aimed to understand the mechanism of H_2_O_2_-producing honeys upon the activation of GOX enzyme.

The objectives of this study were therefore to: (1) investigate substantial synergies arising during the enzymatic conversion of glucose on honey dilution; (2) investigate the bacterial cellular damage and physiological changes caused by the three main stressors in honey; (3) further validate the synergistic effect of model honey components on bacterial mutants of known defective phenotypes.

## Results

### Effect of individual stress factors

Before investigating possible synergies between the three components of model honey, it was necessary to establish the effect of sugars, gluconic acid and H_2_O_2_ as individual stressors on the experimental organism used, *E. coli* K-12 MG1655. Since honey presents its highest antimicrobial activity once diluted, three concentrations of each stressor were chosen to simulate honey dilutions (Table 1: sugars; S70-S30, gluconic acid; G60-G9, H_2_O_2_; H5-H004). Cell viability was determined based on TVC counts and FC was used with the dyes propidium iodide (PI) and bis-oxonol (BOX). PI can only enter cells with a disrupted membrane and BOX enters cells with a collapsed membrane potential ^29^, so the two dyes can be used to differentiate between healthy or intact cells (PI^-^/BOX^-^), injured or depolarized cells (PI^-^/BOX^+^) and cells with disrupted membranes (PI^+^/BOX^+^).

**Table 1:** Composition of model honeys. The “S”, “G” and “H” stands for the sugars, gluconic acid and hydrogen peroxide (H_2_O_2_) respectively.

Model	Components/concentration						
	Sugars (%)	Fructose (M)	Glucose (M)	Maltose (M)	Sucrose (M)	Gluconic acid (mM)	H_2_O_2_ (mM)
S70	70%	1.88	1.56	0.184	0.033	–	–
S50	50%	1.34	1.11	0.131	0.024	–	–
S30	30%	0.8	0.66	0.07	0.014	–	–
G60	–					60	–
G34	–					34	–
G9	–					9	–
H5	–					–	5
H3	–					–	3
H004	–					–	0.04
H1	–					–	1
MSGH	30%	0.8	0.66	0.07	0.014	8.6	0.04
MSG	30%	0.8	0.66	0.07	0.014	8.6	–
MSH	30%	0.8	0.66	0.07	0.014	–	0.04
MGH	–					8.6	0.04
MSGH004	30%	0.8	0.66	0.07	0.014	8.6	0.04
MSGH01	30%	0.8	0.66	0.07	0.014	8.6	0.1
MSGH05	30%	0.8	0.66	0.07	0.014	8.6	0.5
MSGH1	30%	0.8	0.66	0.07	0.014	8.6	1
MSGH3	30%	0.8	0.66	0.07	0.014	8.6	3

The “M” stands for the models combining two or three components in the lowest concentration they were reported on honey diluted between 30 and 70% sugars concentration.

Gluconic acid (1a) and H_2_O_2_ (1b) had a dose-dependent effect on cell viability. Sugars at all tested concentrations decreased the viability of cells by 20% (1c) with no further reduction up to 24 h of treatment (data not shown) and affected the cell size (1d). Although sugars had a bacteriostatic effect, FC showed that up to 40% of sugar-treated bacteria were permeable to both PI and BOX (PI^+^/BOX^+^) (Fig. 2a–c). The most likely interpretation of this is that osmotic stress caused by sugars affected the membrane integrity and caused depolarization. This effect developed more rapidly at treatments with higher sugar concentrations during the first hours of exposure. However, up to 24 h, similar percentages of BOX^+^/PI^+^ cells were observed for all sugar concentrations (Fig. 2c).

**Figure 1:**
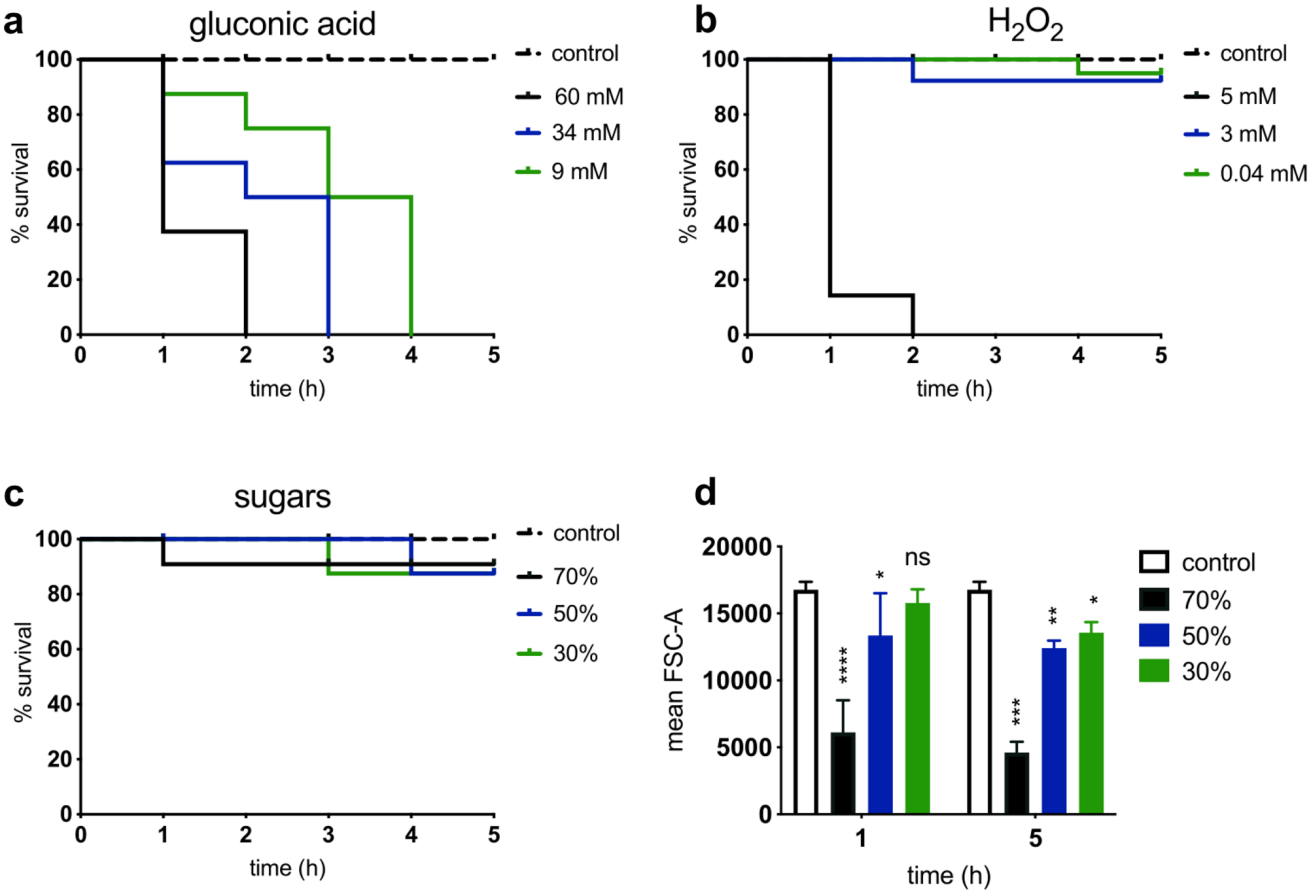
Effects of individual stressors on the viability and phenotype of exponential phase* E. coli* MG1655 cells. (**a–c**) Kaplan–Meier survival plots showing percentage survival post-exposure to increasing concentrations of gluconic acid, H_2_O_2_, and sugars (determined by TVC; detection limit: 20 CFU/mL). (**d**) Changes in mean forward scatter (FSC-A) of bacteria post-challenge with sugar, shows the effect of osmotic stress on bacterial cell size (2-way ANOVA; asterisks show significance levels of Sidak’s multiple comparisons test to the control group (****p < 0.0001, ***p = 0.0003, **p = 0.0028, *p = 0.020 ns; p > 0.05).

Osmotic shock has previously been shown to cause water efflux from cells and eventually decrease the cell volume^30,31^. We therefore determined the effects of different sugar concentrations on average cell size over time, using forward scatter measurements in FC. A substantial decrease in mean forward scatter area (FSC-A) was seen in populations treated with 50% and 70% sugar solutions, with the 70% solution inducing a greater decrease in FSC-A (Fig. 1d). Although osmotic stress caused by sugars generated physiological changes (i.e. depolarization, decrease of cell size, increased membrane permeability) to *E. coli*, cell viability decreased only by 10–20% (Fig. 1c).

**Figure 2:**
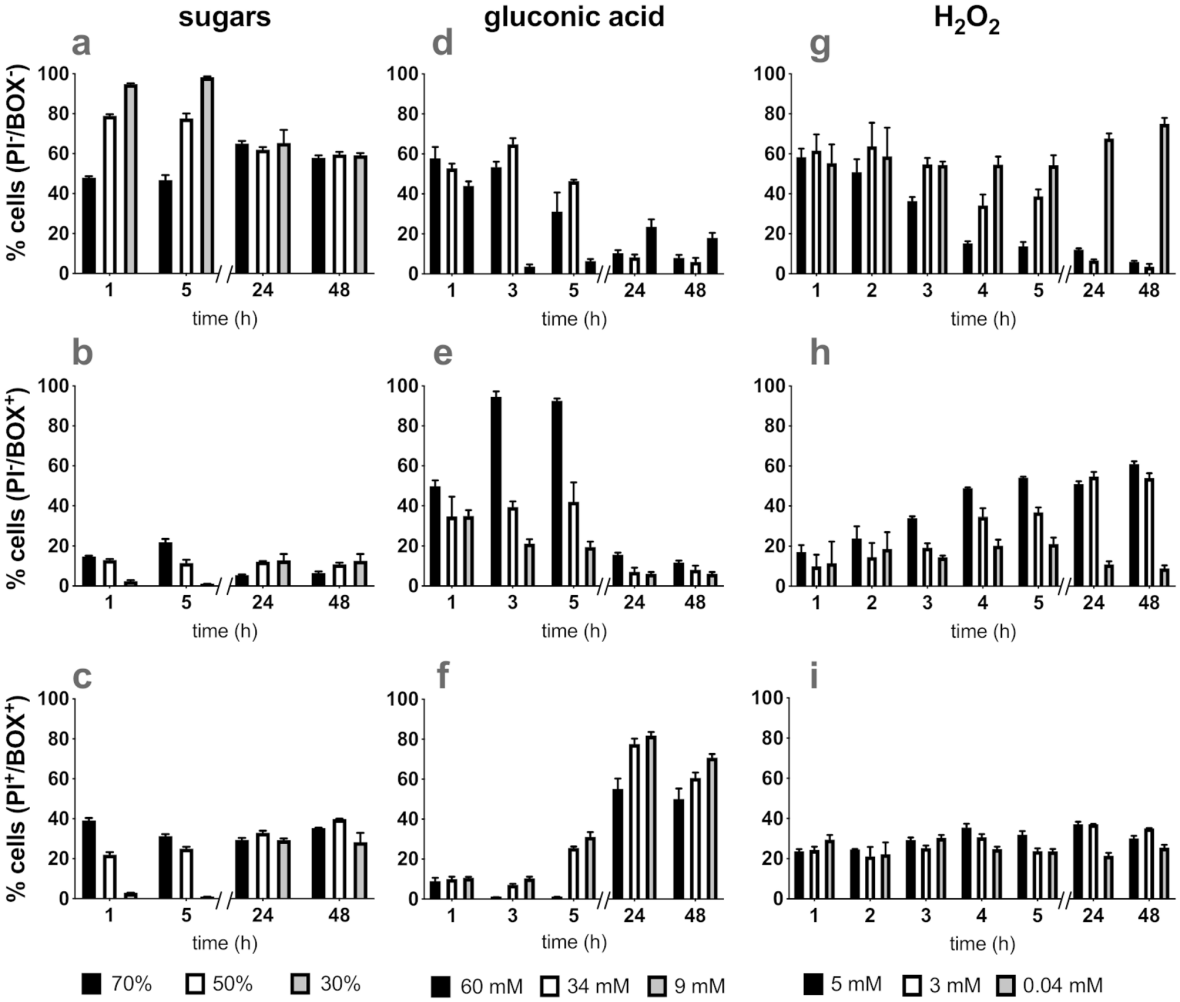
Flow cytometry analysis of *E. coli* MG1655 exposed to single stressors (sugars, gluconic acid and H_2_O_2_) at three levels of concentration. The mean percentage of cells that were (**a**,**d**,**g**) healthy (PI^-^/BOX^-^), (**b**,**e**,**h**) injured or depolarized (BOX^+^/PI^-^), and (**c**,**f**,**i**) dead or membrane damaged (BOX^+^/PI^+^) are shown for bacterial populations exposed to sugars, gluconic acid and H_2_O_2_. Error bars represent the average ± SD (n = 3; biological replicates).

Unlike sugars, both gluconic acid and H_2_O_2_ caused dose-dependent bactericidal effects (Fig. 1a,b). FC showed that gluconic acid decreased the percentage of intact cells in dose-dependent manner (Fig. 2d) while the highest dose of gluconic acid (60 mM) caused an increased percentage of BOX^+^ cells indicative of membrane depolarization (Fig. 2e). Lower doses of gluconic acid caused depolarization (BOX^+^) to a lower extent and gradual membrane permeabilization (BOX^+^/PI^+^) up to 48 h (Fig. 2f). At all concentrations of gluconic acid tested, destruction of membrane integrity developed gradually over time. By 24 h of exposure more than 90% of the population was permeable to PI. For higher dosed cells (60 mM), a lower percentage of cell membrane permeabilization was observed (Fig. 2f). This is might due to the presence of “ghost” cells in which the cytoplasmic content is expelled to the surrounding medium, as a result of the stress applied. These cells might appear as depolarized (BOX^+^/PI^-^) while they do not express the PI fluorescence^32^. Membrane depolarization observed during the first hours of exposure correlated with the loss of cell viability as measured by TVC.

Hydrogen peroxide also had a dose-dependent effect on survival as measured by TVC (Fig. 1b) which was related to the decrease of intact cells as was observed in FC results (Fig. 2g). However, all concentrations of H_2_O_2_ tested caused a similar extent of membrane permeabilization (BOX^+^/PI^+^) (Fig. 2i) while the depolarization effect (BOX^+^) was seen to be dose-dependent and gradually increased over time (Fig. 2h). Thus, the bactericidal effect of H_2_O_2_ was associated with the simultaneous loss of membrane potential and integrity.

Overall, it was concluded that each of the three stressors have clear and distinct effects on bacterial membranes integrity and cell viability and possess distinct mechanisms of killing.

### Synergy between stress factors in model honey

**Figure 3:**
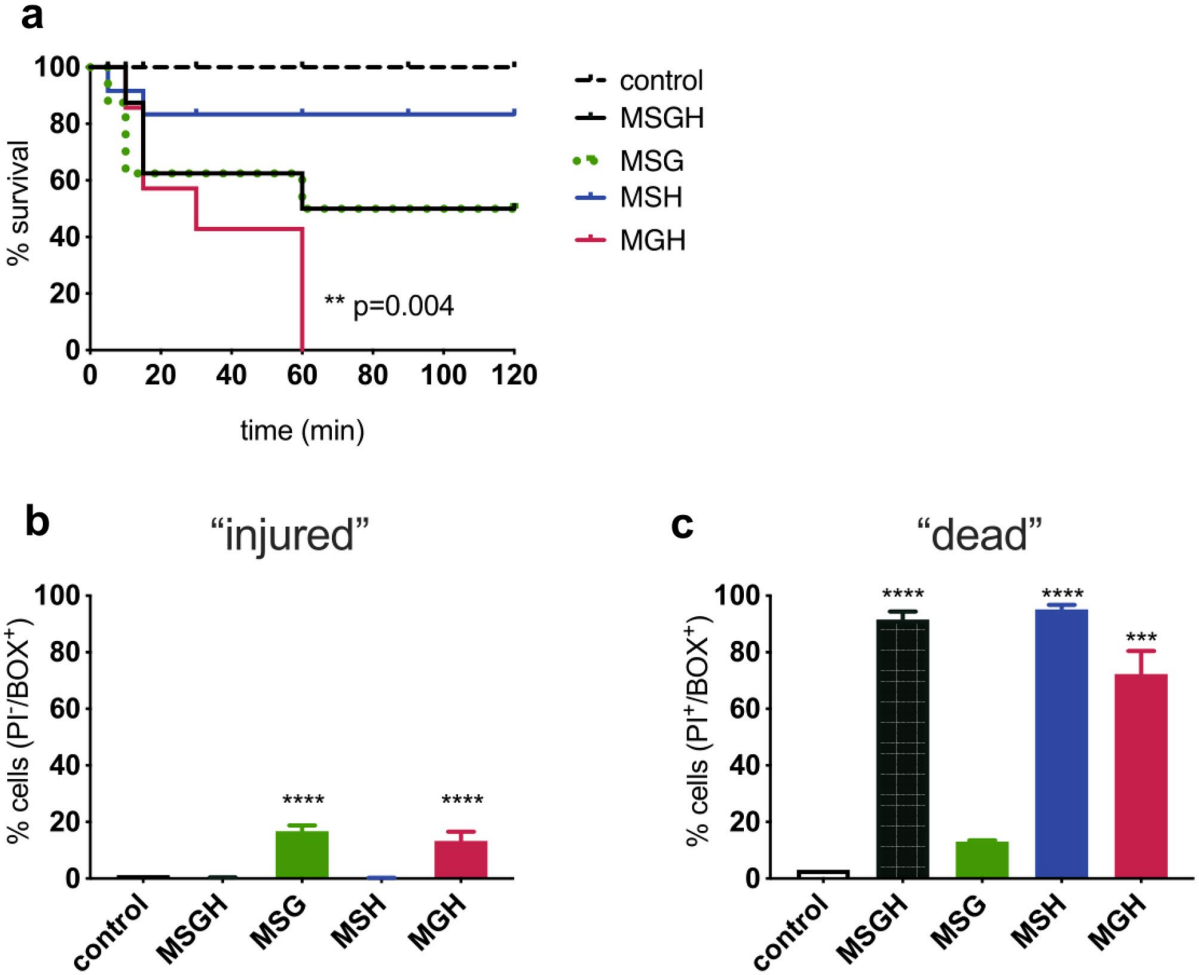
Synergistic effect caused by model honeys on exponential phase *E. coli* MG1655 cells. (**a**) Kaplan–Meier survival plots show percentage survival post-exposure to four model honeys (detection limit: 20 CFU/mL). Survival curves were compared to the control using the Log-rank (Mantel-Cox) test (**; p = 0.004). (**b**,**c**) FC analysis showing the percentage of cells lacking membrane potential (**b**; BOX^+^/PI^-^) and both membrane potential and integrity (**c**; BOX^+^/PI^+^) 2 h post-exposure to 4 model honeys and the control (bacteria in PBS). The significance of relative proportions of BOX^+^/PI^-^ and BOX^+^/PI^+^ between the model-treated bacteria and control was tested with One-way ANOVA (Dunnett's multiple comparisons test; 95% CI; ***p = 0.0001, ****p < 0.0001). Error bars represent the average ± SD (n = 3; biological replicates).

**Figure 4:**
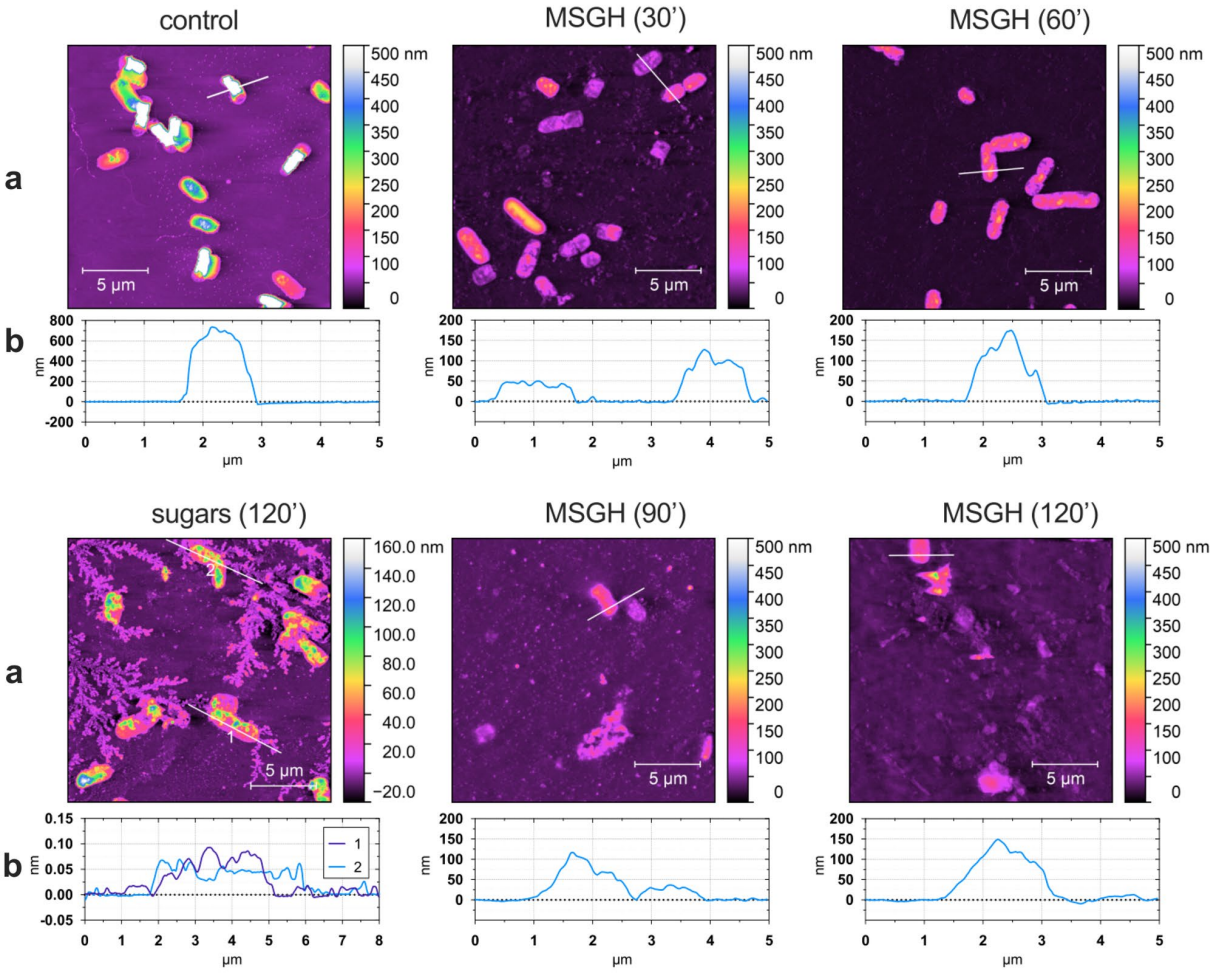
*E. coli* cells topography after 2 h exposure to sugars (S30) and model honey (MSGH). Topography (**a**) and cross section images (**b**) show the effect of model honey (MSGH) and sugars (S30) on surface structure of bacterial cells. Nanoscale structural changes of cross-sectioned bacteria (cells crossed by white line) are given in the graphs below the AFM images. Control was bacteria growing in LB and then transferred in PBS.

To investigate possible synergies between the antimicrobial activities of sugars, gluconic acid and H_2_O_2_, a central composite design (CCD) Response Surface Methodology (RSM) approach was used. RSM was used to optimize the concentration of the components in model honey in order to achieve the highest antibacterial activity towards *E, coli*^33^. This resulted to 12 model honey formulations (see Supplementary Table S1 online). All models caused a rapid bactericidal effect, which made it impossible to locate the optimum concentration of each component for antimicrobial activity (see Supplementary Fig. S1 online). Therefore, subsequently model honeys composed of the three stressors, at the lowest concentrations that have been found on honey dilution (30% sugars, 8.6 mM gluconic acid, 0.04 mM H_2_O_2_), were combined in a model system (MSGH) in order to investigate their synergy. Also, two-component models were used to assess the potential considerable synergies between two of the three stressors (MSG, MSH, MGH). The survival of exponentially-growing *E. coli* MG1655 cells was measured at 30, 60, 90, and 120 min after exposure (Fig. 3a), and changes in bacterial membrane integrity and morphology were detected by FC (Fig. 3b,c) and AFM respectively (Fig. 4).

The results showed that the combination of gluconic acid and H_2_O_2_ (MGH) had the highest antibacterial activity (Fig. 3a). The MGH was the only model that caused simultaneous cell membrane depolarization and permeabilization (Fig. 3b,c), that further induced a significant (p = 0.004) bactericidal effect 1 h post-treatment. This was not observed when sugars were present in the model (MSGH). Measurement of the mean fluorescence intensity (MFI) of PI, an indicator of membrane permeability, revealed that all H_2_O_2_-containing models caused equal or higher cell damage compared to this caused by 250 times more concentrated H_2_O_2_ solution (see Supplementary Fig. S2 online).

Both the MSGH and MSG models demonstrated identical antibacterial activity (50% survival) after 120 min (Fig. 3a), suggesting that H_2_O_2_ at low concentration (0.04 mM) does not have any effect on cell survival in this model honey. However, FC showed significant (p < 0.0001) differences between the cells exposed to MSGH (with H_2_O_2_) and MSG (without H_2_O_2_) (Fig. 3b,c). All H_2_O_2_-containing models (MSGH, MGH, MSH) significantly (p < 0.0001) increased the percentage of PI permeable cells, indicating that the presence of H_2_O_2_ causes some membrane disruption even when present at such a low and non-bactericidal concentration (0.04 mM) (Fig. 3b). This cell damage did not arrest cell growth unless gluconic acid was present (Fig. 3a). Thus, the synergy between low pH and oxidative stress, induced by the gluconic acid and H_2_O_2_ respectively, caused a considerable antimicrobial effect by disrupting membrane polarity and integrity.

AFM images showed morphological alterations of cell structure following the treatment with MSGH (Fig. 4). Within 2 h of treatment the cell height declined from 700 nm (control) to less than 300 nm (MSGH treated sample) as shown by the cross-section analysis. The loss of cellular volume (lower cell height; Fig. 4b) and the surface roughening (Fig. 4a,b), suggests the collapse of the cell wall and potentially the leakage of intracellular material. Likewise, sugar-treated cells were observed to have a roughened surface, and the cell height decreased to 100 nm after 2 h treatment. However, the synergy of all the three stressors caused remarkable cell rupture (such as cell fragments) within 2 h of treatment compared to the sugar solution itself. AFM results agreed with the cell membrane-damaging effects which has been previously observed by FC (Fig. 3b,c).

The investigation using these model honeys revealed that the presence of gluconic acid increases the toxicity of H_2_O_2_ even when the latter is found in low non-inhibitory concentrations (0.04 mM). The synergy of these two components caused simultaneous depolarization of cell membrane and cell wall destruction, two effects which arrested bacterial growth. Although the presence of sugars moderated the toxicity of the other two components, AFM images showed that MSGH model caused major changes to cell structure compared to the sugar solution itself.

### Antibacterial effect of natural honeys

**Figure 5:**
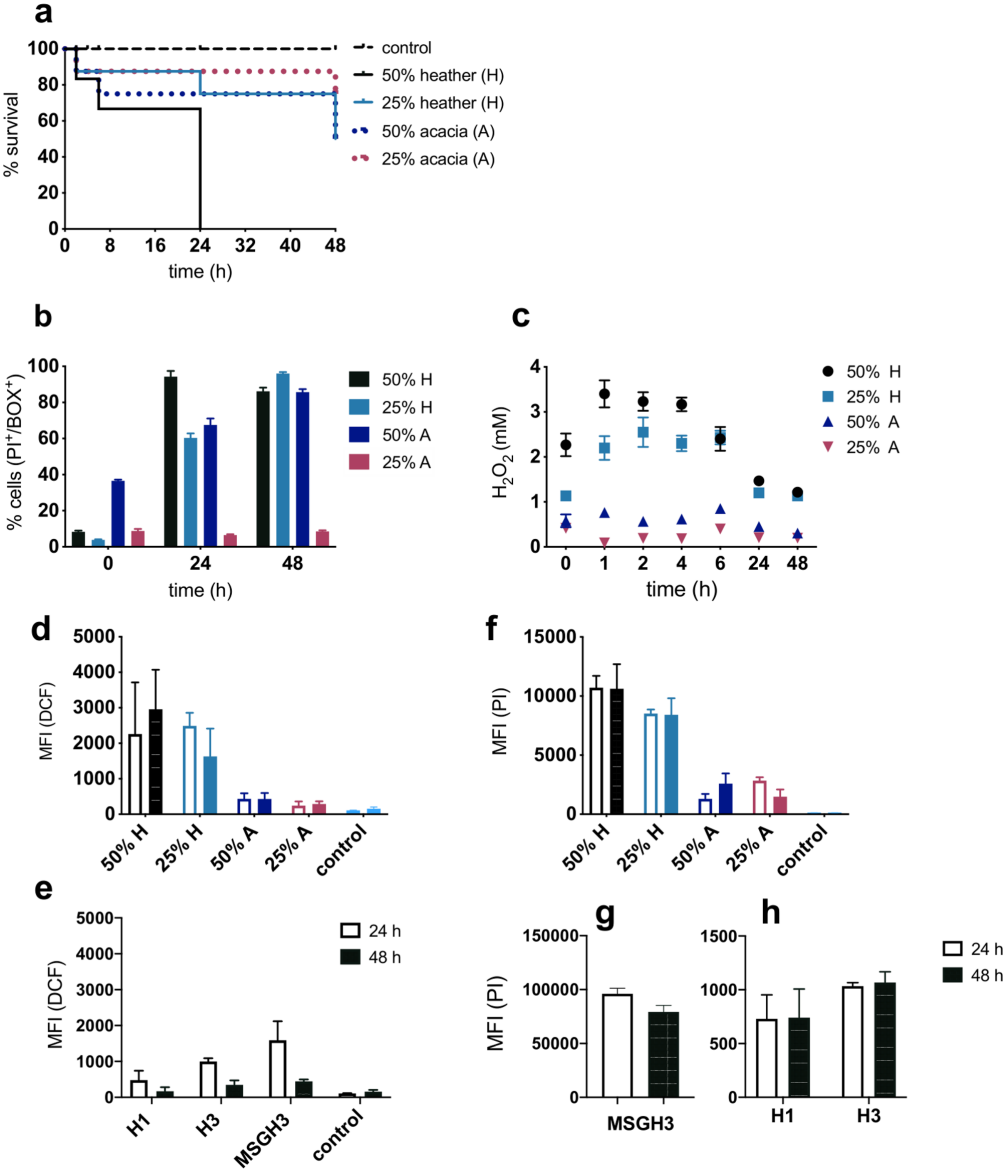
Comparison of antibacterial activity caused by natural and model honeys. (**a**) Kaplan Maier survival plots showing the percentage of *E. coli* MG1655 survival, post-exposure to (“H”) heather and (“A”) acacia honeys, diluted to 50% and 25%, t = 0 corresponds to the initial inoculum. (**b**) FC analysis showing the effect of heather and acacia honey on membrane integrity and potential (% BOX^+^/PI^+^). (**c**) Monitoring of H_2_O_2_ concentration in each honey up to 2 days post-dilution. t = 0 corresponds to a few seconds after the dilution of honey. Mean fluorescence intensity (MFI) of DCF indicates ROS concentration in *E. coli* 24 (open bars) and 48 h (full bars) after exposure to (**d**) heather, acacia honeys and (**e**) model honeys (MSGH3; model honey containing 3 mM H_2_O_2_, H1; H3; H_2_O_2_ solutions containing the maximum H_2_O_2_ found in heather (3 mM) and acacia honey (1 mM)). (**f–h**) The mean fluorescence intensity (MFI) of PI corresponds to the extent of membrane permeabilization post-exposure to the same natural and model honeys. The negative control was cells incubated with PBS. Error bars represent the average ± SD (*n* = 3; biological replicates).

Above, it was shown that H_2_O_2_ and gluconic acid are both involved in the antibacterial effect of the model honeys. H_2_O_2_ and other phytochemical compounds, found in natural honey, have been reported to stimulate the prolonged generation of long-lived and more toxic ROS than the H_2_O_2_ itself^34^. To confirm this, the antibacterial effects of two natural honeys, heather (H) and acacia (A), were tested at a range of dilutions, during which the accumulation of H_2_O_2_ was measured (Fig. 5a–c). ROS-induced oxidative stress was monitored in *E. coli* MG1655 cells treated with natural and model honeys of similar H_2_O_2_ content (MSGH1, MSGH3 containing 1 mM and 3 mM H_2_O_2_ respectively) and H_2_O_2_ solutions (H1 and H3 containing 1 mM and 3 mM respectively) (Table 1).

Results showed the highest antimicrobial activity was demonstrated by honeys diluted at 50% and 25% which accumulated also the highest H_2_O_2_ concentration. That is heather (50%; 3.6 mM), heather (25%; 2.6 mM), and acacia (50%; 0.9 mM) honey (Fig. 5a,c, Supplementary Fig. S3). The above honey concentrations caused bacterial membrane damage and depolarization (PI^+^/BOX^+^) to 87–96% of the cells by 48 h of treatment as determined by FC (Fig. 5b, Supplementary Fig. S4). A similar effect was observed in cells treated with model honeys MGH, MSH and MSHG (Fig. 2b). However, a decrease in the concentration of H_2_O_2_ started within 2 h of the dilution of honey and by 24 h H_2_O_2_ had degraded to non-inhibitory concentration (less than 3 mM) (Fig. 5c). Therefore, to examine whether the killing of *E. coli* was due to other newly formed ROS other than H_2_O_2_, intracellular ROS accumulation was measured by FC with the H_2_DCFDA dye (2′,7′-dichlorodihydrofluorescein diacetate). The results were interpreted based on the mean fluorescence intensity (MFI) expressed by the whole bacterial population when exposed to either natural or model honeys (of equal H_2_O_2_ content). Likewise, for the same populations the MFI of PI was monitored to identify whether the membrane permeabilization is correlated to intracellular ROS accumulation.

Figure 5d shows that intracellular ROS levels in bacteria treated with heather (“H”) were higher than those treated with acacia (“A”) honey. This is in agreement with the H_2_O_2_ levels monitored in each honey 24 h and 48 h post-dilution (Fig. 5c).

Although heather (25%) and acacia (50%) honeys had similar bactericidal effects (Fig. 5a), the former caused higher ROS accumulation within the cells up to 48 h of treatment (Fig. 5d). Similar results were seen in models (H1, H3 and MSGH3) which contain H_2_O_2_ in a range analogous to the natural honeys (1–3 mM H_2_O_2_) (Table 1, Fig. 5e). Model honey (MSGH3), containing 3 mM H_2_O_2_, caused higher ROS levels at 24 h but this declined by 48 h of treatment in contrast to natural honey. Bacterial membrane permeabilization (MFI of PI), post-exposure to natural honeys, paralleled the accumulation of ROS (Fig. 5f). Bacteria exposed to model and natural honeys (Fig. 5f,g) expressed considerably higher (100 times) PI fluorescence compared to those exposed to H_2_O_2_ solution (H1 and H3) (Fig. 5h). This agrees with our previous results which showed that the combination of the stressors in the model caused higher cell damage comparing to the respective H_2_O_2_ solutions (see Supplementary Fig. S2 online).

These results suggest that H_2_O_2_ activity of natural honeys is a determinant of the ROS-inducing effect. Regardless the decrease in the concentration of H_2_O_2_ soon after its generation, it was seen that its generation triggers the production of other ROS with long-lived toxicity. It also appears that it is the synergy of honey stressors, rather than the H_2_O_2_-activity itself, that causes an oxidative-like bacterial stress. This is consistent with the observations made with the model honeys.

### Use of catalase-depleted *E. coli* mutants to confirm the role of oxidative stress in the antibacterial activity of model honey

**Figure 6:**
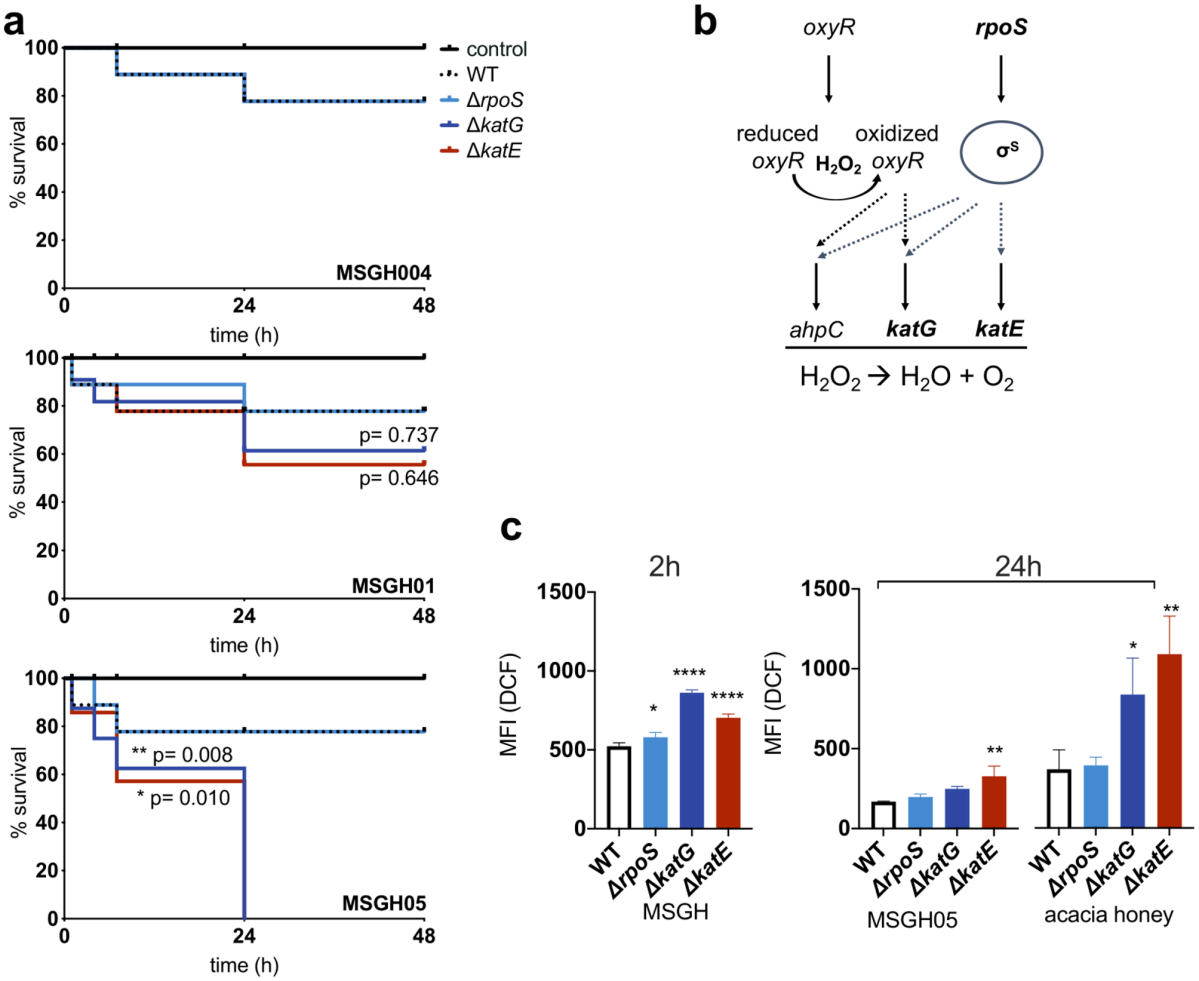
Identification of H_2_O_2_-induced oxidative stress in honey by comparing the susceptibility of catalase deficient (*katG*, *katE*) and *rpoS* mutants. (**a**) Kaplan Maier survival plots show survival of the knockouts and the WT to model honeys of increasing H_2_O_2_ level. Survival curves were compared to the WT with the Log-rank (Mantel-Cox) test (*p = 0.02, **p = 0.008). (**b**) Model for the antioxidant defense mechanism in *E. coli* regulated by *oxyR* and *rpoS.* (**c**) Mean fluorescence intensity (MFI) of DCF indicates the ROS accumulation, in mutants and the WT, 2 h and 24 h post exposure to model and real honey (25% acacia, 0.5 mM H_2_O_2_). One-way ANOVA test was used to compare the significance in ROS accumulation between the WT and the mutants (Δ*katG*, Δ*katE*) and Δ*rpoS* Dunnett’s multiple comparison test (95% CI, significance p < 0.05), model honey (2 h) (*p = 0.0465, ****p < 0,0001), model honey (24 h) (*p = 0,0298, **p = 0,0028), acacia (24 h) (**p = 0,0013). Error bars represent the mean ± SD (*n* = 3; biological replicates).

As seen in previous sections, the bactericidal strength of honey is strongly affected by the available H_2_O_2_ which further stimulates the generation of ROS in a concentration-dependent manner. It was also demonstrated that the presence of gluconic acid and sugars is essential for the antibacterial activity of honey. However, bacteria employ ROS scavenging and DNA repair mechanisms to overcome the oxidative stress caused by ROS^35^. In *E. coli*, general stress responses are modulated by regulators such as the sigma factor RpoS, while responses to oxidative stress are regulated by OxyR and two component system SoxR/SoxS^35,36^. Hydroperoxidase I (encoded by *katG*) is transcriptionally induced by OxyR in exponentially growing cells in the presence of low H_2_O_2_, or by RpoS, in an OxyR-independent manner. The expression of Hydroperoxidase II (encoded by *katE*) is controlled by RpoS. Both *katG* and *katE* genes catalyse the breakdown of H_2_O_2_ to water and oxygen^37,38^ (Fig. 6b). We therefore tested whether oxidative stress is the predominant honey stressor by determining the susceptibility of strains lacking catalase genes (Δ*katG* and Δ*katE*) or the general stress response regulator (Δ*rpoS*), to model honeys with increasing H_2_O_2_ content (MGH004, MGH01, MGH05; containing 0.04, 0.1 and 0.5 mM H_2_O_2_) (Table 1). The susceptibility of the mutants was compared to that of the WT.

As shown in Fig. 6a, both Δ*katG* and Δ*katE* were impaired in their ability to survive when compared to the Δ*rpoS* strain and the WT. These strains were equally susceptible to all three models tested (MGH004, MGH01, MGH05). Physiological changes monitored by FC showed that the 3 model honeys caused the same extent of membrane damage to mutants and the WT. The model with lower H_2_O_2_ concentration (MSGH004) caused a gradual increase of cells with damaged membranes while the others (MSGH01, MSGH05) caused membrane depolarization and damage soon after the treatment. Although detoxifying mechanisms in the WT can degrade the H_2_O_2_ it was shown that presence of this compound induced immediate cell damage to all 4 strains during the first minutes of exposure (see Supplementary Fig. S5 online).

ROS accumulation was measured within the four strains after exposure to the three model honeys. Figure 6c shows that ROS content was significantly higher (p < 0.0001) in both catalase mutants 2 h after exposure to model honey (MSGH05) compared to the WT and the Δ*rpoS*. However, the Δ*katE* accumulated significantly (p = 0.008) higher ROS up to 24 h of exposure to MSGH05. Accumulation of ROS in four strains was also tested after exposure to acacia honey which has equal H_2_O_2_ activity to the MSGH05. Both Δ*katG* and Δ*katE* were seen to accumulate significantly higher ROS (Δ*katG* p = 0.0028; Δ*katE* p = 0.0013) compared to the Δ*rpoS* and the WT (Fig. 6c). In contrast to model honey, acacia stimulated the prolonged production of ROS up to 24 h after treatment.

These results confirmed that H_2_O_2_ is one of the main stressors in honey since catalase-depleted mutants demonstrated increased susceptibility compared to the WT and Δ*rpoS*. However, the phenotype of both WT and Δ*rpoS* confirmed the synergy underlying (model) honey that demonstrates antibacterial activity even at non-bactericidal H_2_O_2_ concentration. This is possibly due to the coupling of H_2_O_2_ with the gluconic acid that simulates the production of ROS that further cause an oxidative-like stress effect.

## Discussion

In this study, the use of a model honey, comprising the three main microbial stressors, allowed the identification of previously unknown synergies in the antibacterial activity of H_2_O_2_-producing honeys. The combination of phenotypic profiling with morphological analysis of *E. coli*, exposed to model and natural honeys, revealed likely cell targets, and provided a potential explanation for the underlying antibacterial mechanism.

The synergy of H_2_O_2_ and gluconic acid was significant. Both components affected the polarity of the membrane and the integrity of the cell wall. FC analysis showed that each of the components caused distinct time- and dose-dependent physiological changes critical for bacterial viability. Gluconic acid caused membrane depolarization and prolonged exposure to this weak acid caused membrane destruction. This may be explained by the interference of the undissociated acid with the membrane proteins and the progressive destruction of the cell envelope, as was reported previously^39^. In contrast, H_2_O_2_ caused simultaneous cell wall destruction and membrane depolarization, the latter of which was proportional to the dose applied. According to previous studies, H_2_O_2_ at concentrations as low as 30 μM, diffuses freely through bacterial membranes initiating the oxidation of lipid molecules which progressively causes damage^40^. The diffusion of radicals through bacterial cell wall and the peroxidation of cell membrane lipids compromises the membrane potential and destructs the cell wall integrity^41^. This explains the cell wall destruction observed in all bacterial populations post-exposure to H_2_O_2_-containing models and natural honeys. The synergy of the two stressors (H_2_O_2_ and gluconic acid) caused a simultaneous effect on membrane potential and integrity. These phenotypes became visible soon after the exposure of *E. coli* to the model honey and were followed by a rapid decrease of the TVC. The increased toxicity of H_2_O_2_ in the presence of low pH (due to the gluconic acid) has already been reported^42^. It is likely that presence of weak acid accelerates the production of ROS^43^. Although the mechanistic explanation of this is not known, it explains the fact that the H_2_O_2_ in honey can be effective as an antibacterial even if it accumulates at concentrations lower than minimum bactericidal concentrations (0.8–8 M)^44^.

The presence of sugars moderated the toxicity of gluconic acid and H_2_O_2_. This is potentially due to the breakdown of sugars by bacteria to extract energy. As has been reported, sugar concentration up to 80% is bactericidal while at lower concentration (40% or less) bacteria breakdown di-, oligo- or polysaccharides to form monosaccharides and further lactate^45^. Thus, we speculate that the presence of sugars counteracts to some extent the effect of acid/oxidative stress. However, FC showed that sugars, at a concentration up to 50%, caused a significant (p < 0.0001) decrease of the cell size. This phenotype is associated to plasmolysis and the increased cell membrane permeability which has been observed as a result of osmotic stress. This is potentially due to the separation of the outer from the inner membrane and the collapse of the cell structure^47,48^. Although osmotic upshift caused considerable phenotypic changes, none of the concentrations tested arrested the cells’ growth. This suggests that sugars may contribute to the antibacterial effect by causing alterations to the physiology of cells. However, the presence of sugars moderates the toxicity caused by the gluconic acid and H_2_O_2_. This agrees with the fact that honey can be bactericidal yet non-toxic for human cells when this is applied to open wounds and ulcers^48^.

The effects of the model honey were confirmed by heather and acacia. Similarly, to the model honey diluted natural honeys, which yielded higher than 1 mM of H_2_O_2_, caused membrane depolarization and cell wall destruction. However, only honeys accumulated up to 2 mM H_2_O_2_ arrested cell viability. This suggests that low H_2_O_2_ (less than 1 mM) only causes transient bacterial damage. This is might due to the catalase activity of *E. coli* that degrades the H_2_O_2_. In this event, it is possible that cells can repair the damage caused by oxidative stress and resume growth using the available carbon sources of honey sugars. In contrast, for higher H_2_O_2_ concentration is possible that catalase is insufficient to remove the peroxide, and hence that this higher level of H_2_O_2_ causes prolonged oxidative damage^35^. The oxidative damage can be enhanced by the Fenton reaction which produces more reactive and toxic radical species within honey^49^. According to previous findings, the polyphenols and transition metals (i.e. Cu(I), Fe(II)) found in honey, serve as intermediates of Fenton reaction and enhance the ROS generation^15,35^. Our results showed a quick degradation of H_2_O_2_. However, the prolonged exposure of bacteria to honeys with higher peroxide activity increased the intracellular accumulation of ROS. Heather honey (dark colour) stimulated higher ROS generation comparing to acacia (light colour). This is might be related to the abundance of polyphenols in darker honeys. However, since the composition of both honeys is uncharacterized, further conclusions could not be made.

These results suggest that the antimicrobial activity of honey is highly dependent on the availability of H_2_O_2_ which further triggers the ROS production. Also, in this study we demonstrated that low pH and potentially high osmolality enhance the toxicity of H_2_O_2_ by causing severe damage on membrane polarity and integrity.

The increased susceptibility of strains lacking catalase (Δ*katG* and Δ*katE*) to model honeys of increasing H_2_O_2_ confirmed the effect of H_2_O_2_-induced oxidative stress in honey. As shown in a previous study, the H_2_O_2_ concentration is controlled by the balance between H_2_O_2_ production and its degradation by AhpC (78%), catalase (12%) and membrane permeability (10%) that serves as a passive defence against H_2_O_2_^50^. However, for exogenous H_2_O_2_ higher than 30 μΜ, only catalase activity can defend bacteria from oxidative stress^38^. In agreement with this, here it was seen that catalase mutants were unable to detoxify the H_2_O_2_ and eventually lost viability. In contrast, a strain deleted for *rpoS* showed similar sensitivity to the WT. This might be explained either by the relatively low expression of *rpoS* in exponentially growing cells or by the existence of OxyR regulon which activates *katG* and *ahpC* for the detoxification of H_2_O_2_ in cells in exponential growth phase^40^. Here, it was seen that Δ*katE*, the RpoS-transcribed catalase, had a significant growth defect at increasing H_2_O_2_ concentration. Therefore, it was assumed that *rpoS* exerts an activity in exponentially growing cells, while in Δ*rpoS* strains the presence of OxyR protects cells from oxidative stress.

Further, FC analysis showed that increased H_2_O_2_ caused rapid membrane destruction in all strains regardless of their catalase activity. This is probably explained by the ease of H_2_O_2_ diffusion within the bacterial cell which can further cause oxidative damage unless is degraded by the bacterial detoxifying mechanism^50^. Here we showed that the detoxifying activity in the *rpoS* mutant and in WT made cells more resistant towards the treatment with model honeys of increasing H_2_O_2_ content^51^. However, the phenotype of both strains (WT and Δ*rpoS*) implies that the synergy between the main honey stressors induces antibacterial effect that becomes more potent whereas H_2_O_2_ is more abundant. This is due to the longer lifetime of H_2_O_2_, within the cell, that triggers the formation of other ROS species.

Overall, in this study, we have developed a method that uses a model system to allow the evaluation of synergies occurring upon dilution of H_2_O_2_-producing honeys. Although the model system presents some limitations over the real honey, it provides the advantage to understand the fundamental antibacterial mechanism of all H_2_O_2_-producing honeys regardless the species-related variability. For the first time, a well-defined link has been established between the synergies of honey stressors, arising from glucose oxidation, and its antibacterial mechanism. Reproducibility of the individual biological replicates shows that both flow cytometry and TVC methods are accurate and sensitive to input data (see Supplementary Fig. S6 online). Our data suggest that synergy between gluconic acid and H_2_O_2_ is responsible for most of the antibacterial activity in honey. Their synergy caused an oxidative-like bacterial damage which was triggered by the presence of higher H_2_O_2_ concentration in the respective model honeys. This effect caused cell-wall damage and prolonged accumulation of intracellular ROS that eventually arrested cells’ viability.

Since oxidation of glucose was seen to play a key role in the antimicrobial potency of honey, further research should be focused on understanding this reaction, and on the factors that control prolonged ROS generation during enzymatic conversion of glucose. The knowledge of the mechanistic action of honey and the bacterial cell responses to this treatment will provide a basis for the design and formulation of honey-based medical products of enhanced antibacterial activity. Together, this should hasten the wider acceptance of honey as an alternative antimicrobial.

## Methods

### Strains and growth conditions

*E. coli* K-12 MG1655 and derivatives thereof were used for all the experiments. Deletions in genes encoding catalase marked with a kanamycin resistance gene (Δ*katG*::kan and Δ*katE*::kan) and the stress sigma factor RpoS (Δ*rpoS*::kan) were constructed by P1 transduction from the Keio library and validated using PCR with appropriate gene-specific primers (supplementary Table S2) as described previously^52^. Bacteria were grown overnight in 5 ml of Luria Broth (LB; tryptone (10% w/v), yeast extract (5% w/v), sodium chloride (10% w/v)) (Sigma-Aldrich., UK) in 20 ml conical flasks, shaken at 150 rpm at 37 °C. Overnight cultures were diluted in fresh LB to A_600nm_ 0.005 and incubated until the A_600nm_ reached 0.5 McFarland Standard (approx. 10^8^–10^9^ cfu/ml). Before use, cells were pelleted (3900 g for 3 min in an Eppendorf Centrifuge 5810), washed twice in PBS (Oxoid Ltd., UK), and resuspended in PBS to a final absorbance of 0.5.

### Model honey and natural honey samples

Acacia and heather honey (examples of light and dark colour honeys respectively) were purchased from a local retailer (Birmingham, UK). Honey samples were stored in their original packaging, at room temperature (22 °C) in the dark. Fresh solutions of serially diluted honey in deionized sterile water were prepared in concentrations of 50, 25, 12, 6, 3 and 1.5%.

The stock model honey was prepared by dissolving fructose (2.24 M), glucose (1.85 M), maltose (0.219 M) and sucrose (0.04 M) (all purchased from Sigma-Aldrich., UK) in deionized sterile water at 37 °C as described previously^53^. The osmolality was measured at room temperature using a refractometer (Master-Honey/BX, Atago). Stock solutions of gluconic acid and H_2_O_2_ (Sigma-Aldrich., UK) were prepared in deionized sterile water and added immediately before the start of each assay. The compositions of the model honeys used in the antibacterial assays, as they were made from the initial stock, are presented in Table 1.

### Antibacterial assay

The antibacterial activities of natural and model honeys were determined using total viable counts (TVC) and flow cytometry (FC). Natural honey (diluted between 50–1.5%), model honey (MSGH, MSG, MSH, MGH), solutions of H_2_O_2_ (H5-H004), gluconic acid (G60-G08), sugar (S70-S30) (Table 1) and PBS (negative control) were inoculated with exponential phase cultures (~ 10^8^ cfu/ml) at a 1:1 (v/v) ratio (100 μl total volume).

### Total viable counts (TVC)

Samples were incubated at room temperature (22 °C), for the respective time intervals (30, 60, 90, 120 min and 1–48 h), and bacterial counts were measured by serial tenfold dilution in sterile PBS. Five μl of each dilution were spotted into square LB agar (Greiner Bio One, UK) plates. The plates were then tilted to spread the spot into a line down the plate. Colonies were counted after 20 h incubation at 30 °C (this temperature was selected after optimization of the protocol by comparison of 37 °C and 30 °C, to obtain distinct colonies). Survival was expressed as a percentage of the untreated culture (control) using the Kaplan–Meier survival analysis. The lower detection limit of this assay was 20 cfu/ml.

### Flow cytometry

FC analysis was conducted using a BD Accuri C6 flow cytometer (Becton Dickinson Biosciences, Oxford, UK). Samples (10^8^ cells) taken at appropriate time intervals were diluted with 0.2 µm-filtered PBS. For all assays, samples were excited using a 488 nm solid-state laser. 25,000 data points were collected at a maximum rate of 2,500 events/sec and the data were analysed using CFlow (BD) software. Forward scatter area (FSC-A) is proportional to cell size and side scatter area (SSC-A) is an indicator of cellular internal complexity or granularity. For analysis of membrane permeability and potential, samples were stained directly with 4 μg/ml propidium iodide (PI) and 2 μg/ml bis-(1,3-dibutylbarbituric acid) trimethine oxonol (BOX, also called DiBAC_4_(3)) (Sigma, UK) and incubated at room temperature for 10 min in the dark prior to analysis. Untreated bacteria and bacteria treated with 3 M H_2_O_2_ for 30 min, served as “healthy” and “dead” controls, respectively. Fluorescence was detected using 533/30 BP and 670 LP filters corresponding to BOX and PI fluorescence, respectively. Intracellular ROS accumulation was measured with 2′,7′-dichlorofluorescein diacetate (H_2_DCFDA) (Sigma- Aldrich, UK). Cell suspensions (10^8^ cfu/ml) in PBS were treated with model honey and incubated at room temperature for 2 h and samples were taken at 15, 30, 60, 90, and 120 min post-exposure. Treated cells were centrifuged and washed in PBS and incubated with H_2_DCFDA (10 μl/ml) for 1 h at 37 °C in the dark. Samples were washed and the pellet was resuspended in prewarmed PBS. Untreated cells maintained in PBS served as a negative control. Fluorescence was measured using a 533/30 BP filter.

### Hydrogen peroxide assay

Hydrogen peroxide concentration was determined using the Fluorimetric H_2_O_2_ assay kit (Sigma-Aldrich, UK), according to the manufacturer’s instructions. The red fluorescence formed after the reaction of peroxidase and H_2_O_2_ was measured at 540 nm excitation and at 590 nm emission using the CLARIOstar (BMG Labtech, US) multi-detection microplate reader. Dose–response curves were generated using the MARS software (BMG LABTECH, US). To calculate the H_2_O_2_ concentrations in different honeys, a standard curve was generated (see Supplementary Fig. S7 online) using dilutions of a fresh 20 mM H_2_O_2_ stock solution. All determinations were performed in triplicates.

### Atomic force microscopy (AFM)

AFM images of treated bacterial samples were acquired with a Bruker Innova in dry condition on a ~ 25 mm^2^ p-type silicon wafer (Sigma-Aldrich). Before depositing bacterial suspension onto the substrate, it was cleaned with a CO_2_ snow jet while being held on a hot surface at 300 °C. Subsequently, 5 μl of *E. coli* (10^6^ cfu/ml) suspended in PBS were deposited onto the substrate and allowed to air dry. Before scanning the sample, the substrate was rinsed with deionized water to reduce build-up of salt during dehydration. The images were acquired in tapping mode with a BRUKER RTESPA-300 probe (T: 3.4 μm; L: 125 μm; W: 40 μm; f_0_: 300 kHz; k: 40 Nm^−1^).

### Response surface methodology

A Response surface methodology (RSM) design of experiments approach and central composite design (CCD) was used to evaluate the significance of the interaction between the three explanatory variables (honey stressors; sugars, gluconic acids, H_2_O_2_) and the response variable (antimicrobial activity)^54^. RSM was used in order to locate the optimum concentration of the three stressors (gluconic acid, sugars and H_2_O_2_) for the model to achieve the highest antibacterial activity. A central composite design (CCD), as was generated by JMP software (version 7.0, Statistical Discovery^TM^, SAS Institute), studied the three explanatory variables into five different levels. Each level represents a concentration, for each explanatory variable, within the desirable range. According to the design, 12 experiments were conducted containing three replicates for estimating the experimental uncertainty (see Supplementary Table S1 online).

### Experimental design and statistical analysis

The exact sample size (n) for each experiment is given in the respective figure legends. Data are mean ± SD of the independent experiments and are expressed as individual data points and mean ± SD. The statistical analysis and graphical display were performed in GraphPad Prism (https://www.graphpad.com). Survival distributions, within different bacteria treatments, were compared with two-way ANOVA (Sidak’s multiple comparisons test). Statistical testing of differences from three data groups or more was performed using one-way ANOVA followed by Dunnett's multiple comparisons test. All P values and significance levels are indicated in the figures and figures legends.

## Supplementary information

Supplementary Information.

## Data Availability

All data generated or analysed during this study are included in this published article (and its Supplementary Information files).
