# Supplementary material for: Use of a model to understand the synergies underlying the antibacterial mechanism of H2O2-producing honeys
Source: Sci Rep. 2020 Oct 19;10:17692. doi: 10.1038/s41598-020-74937-6 (PMC7573686; doi:10.1038/s41598-020-74937-6)
Supplement: Supplementary file 1 — Supplementary Information. [file 41598_2020_74937_MOESM1_ESM.pdf]

Use of a model to understand the synergies underlying the antibacterial mechanism of H_2O_2-producing honeys

Maria Masoura[1,2], Paolo Passaretti[1], Tim W. Overton[1], Pete A. Lund[2], Kostas Gkatzionis[1,3*]

[1]School of Chemical Engineering, University of Birmingham, Birmingham, B152SA, UK
[2]Institute of Microbiology and Infection (IMI), University of Birmingham, Birmingham, B152SA UK
[3]Department of Food Science and Nutrition, School of the Environment, University of the Aegean, Lemnos, Greece

*** Corresponding author:**
Kostas Gkatzionis K.Gkatzionis@bham.ac.uk

Supplementary Table S1. Composition of the model honeys as proposed by CCD (Central Composite Design) experiment.

	Components' concentration			Individual sugar concentrations			
TEST	Gluconic acid (mM)	H_2O_2 (mM)	Sugars (%)	Fructose (M)	Glucose (M)	Maltose (M)	Sucrose (M)
1	34.3	0.6	62.5	1.675	1.387	0.163	0.029
2	8.6	4.7	62.5	1.675	1.387	0.163	0.029
3	34.3	4.7	62.5	1.675	1.387	0.163	0.029
4	34.3	4.7	70	1.876	1.554	0.183	0.032
5	49.6	7.1	67	1.795	1.487	0.175	0.031
6	60	4.7	62.5	1.675	1.387	0.163	0.029
7	19	7.1	58	1.554	1.288	0.152	0.027
8	49.6	2.26	67	1.795	1.487	0.175	0.031
9	19	2.26	67	1.795	1.487	0.175	0.031
10	49.6	7.1	58	1.554	1.288	0.152	0.027
11	34.3	4.7	55	0.855	0.708	0.084	0.015
12	34.3	8.8	62.5	1.675	1.387	0.163	0.029

Supplementary Table S2. Gene primers designed for the validation of P1 transduction of mutants from *E. coli* BW25113 to MG1655 strain.

Gene name	primer
katG (F)	5'- TGCCCGTTCCATCAGG -3'
katG (R)	5'- TACAGCAGGTCGAAACGG -3'
katE (F)	5'- ATGTCGCAACATAACGAAAAGAACC -3'
katE (R)	5'- TCAGGCAGGAATTTTGTCAATCTTAG -3'
rpoS (F)	5'- TATCGAGGCAGCAAAGGACAGG – 3'
rpoS (F)	5'- GGTGCGTATGGGCGGTAATTTGACC- 3'

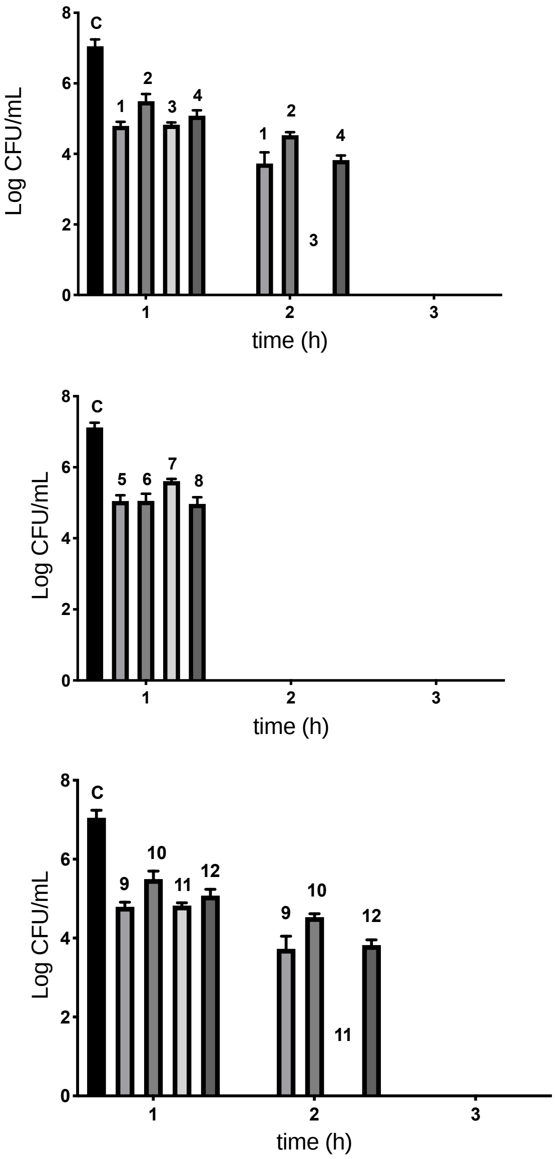

Supplementary Figure S1. Susceptibility of exponentially growing *E. coli* K-12 to model honeys defined by the RSM experiment (Supplementary Table 2). Antibacterial assay was conducted up to 48 h. However, bacterial viability was lower than the detection limit (20 cfu/ml) soon after exposure. No resuscitation was reported for any of the challenged bacterial populations shown above. Error bars represent the average ± SD (n=3; biological replicates).

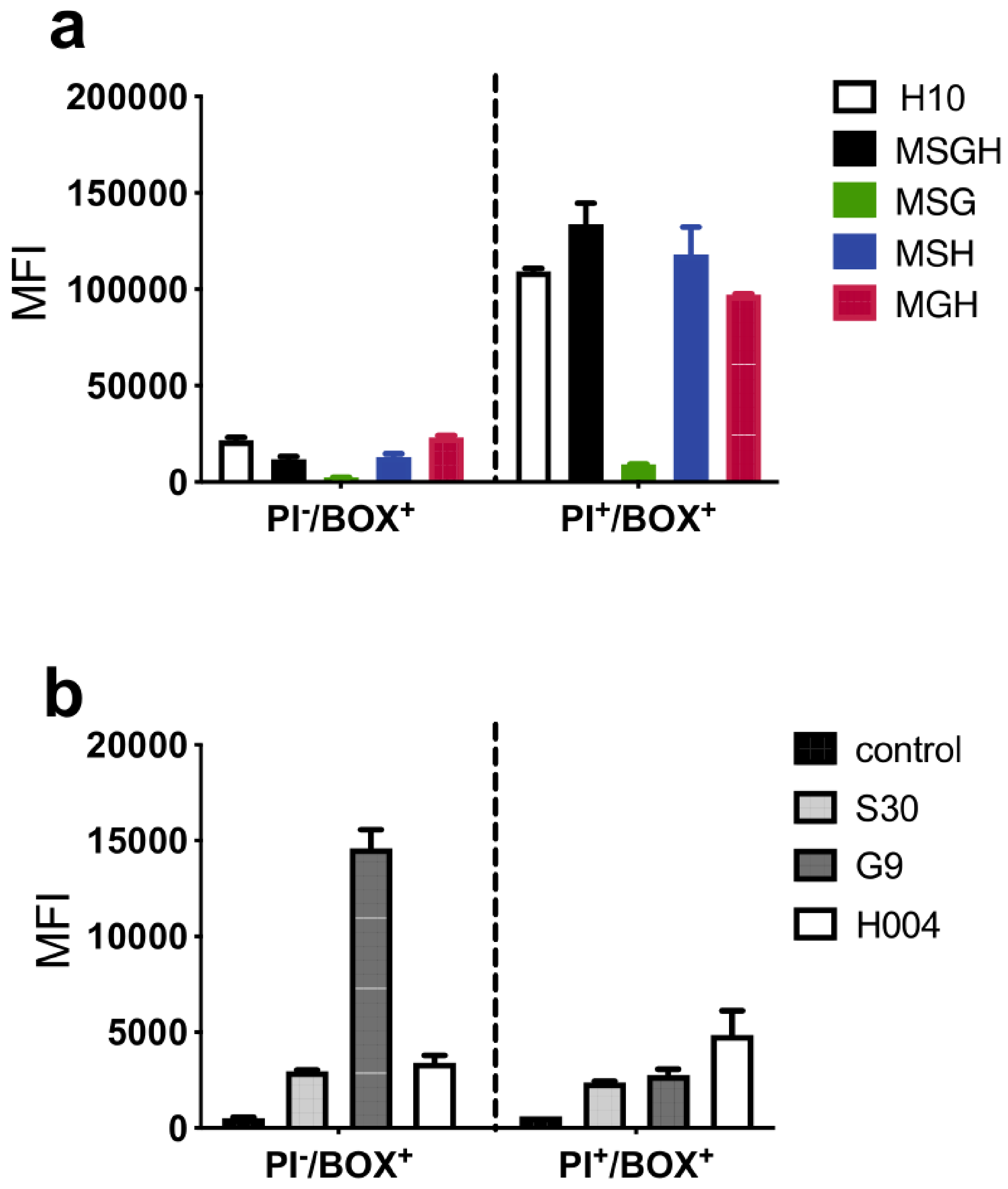

Supplementary Figure S2. Comparison of the effect caused by model honeys and single honey stressors. Mean Fluorescence Intensity (MFI) of (**a**) cells exposed to model honeys and pure H_2O_2 (10 mM) and (**b**) to single stressor models highlights the effect of synergy over the effect of the single stressors on membrane depolarization (BOX^+/PI^-) and integrity (BOX^+/PI^+). Error bars represent the average ± SD. (n=3; biological replicates).

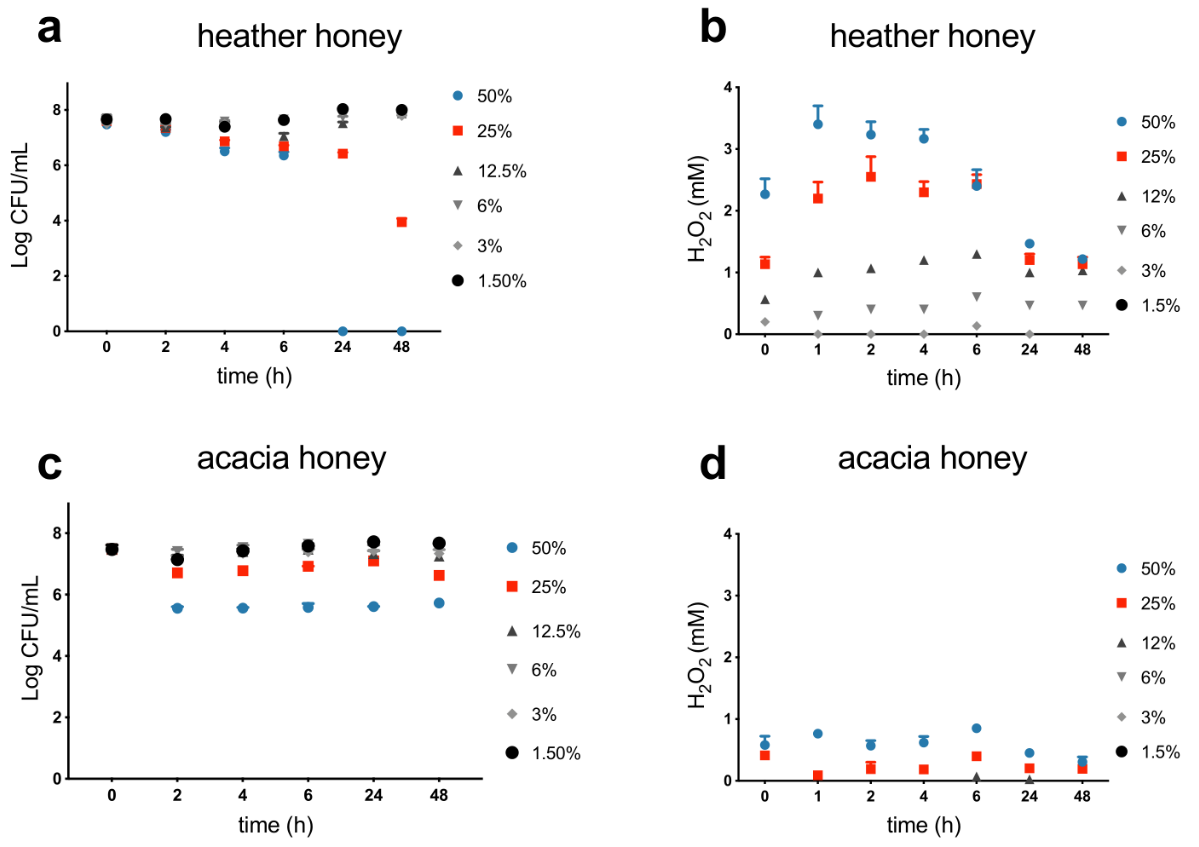

Supplementary Figure S3. Correlation between antimicrobial strength of honey and H_2O_2 accumulation. (**a**) Heather and (**c**) acacia honey were diluted (50-1.5%) and tested for their antimicrobial activity on *E. coli* up to 48 hours of treatment. (**b, d**) The H_2O_2 accumulation was measured for the same time course in order to identify the correlation between the antimicrobial effect of honey and the kinetics of H_2O_2. Error bars represent the average ± SD of three (n=3) biological replicates and 3 individual measurements of H_2O_2 accumulation in the respective honey samples.

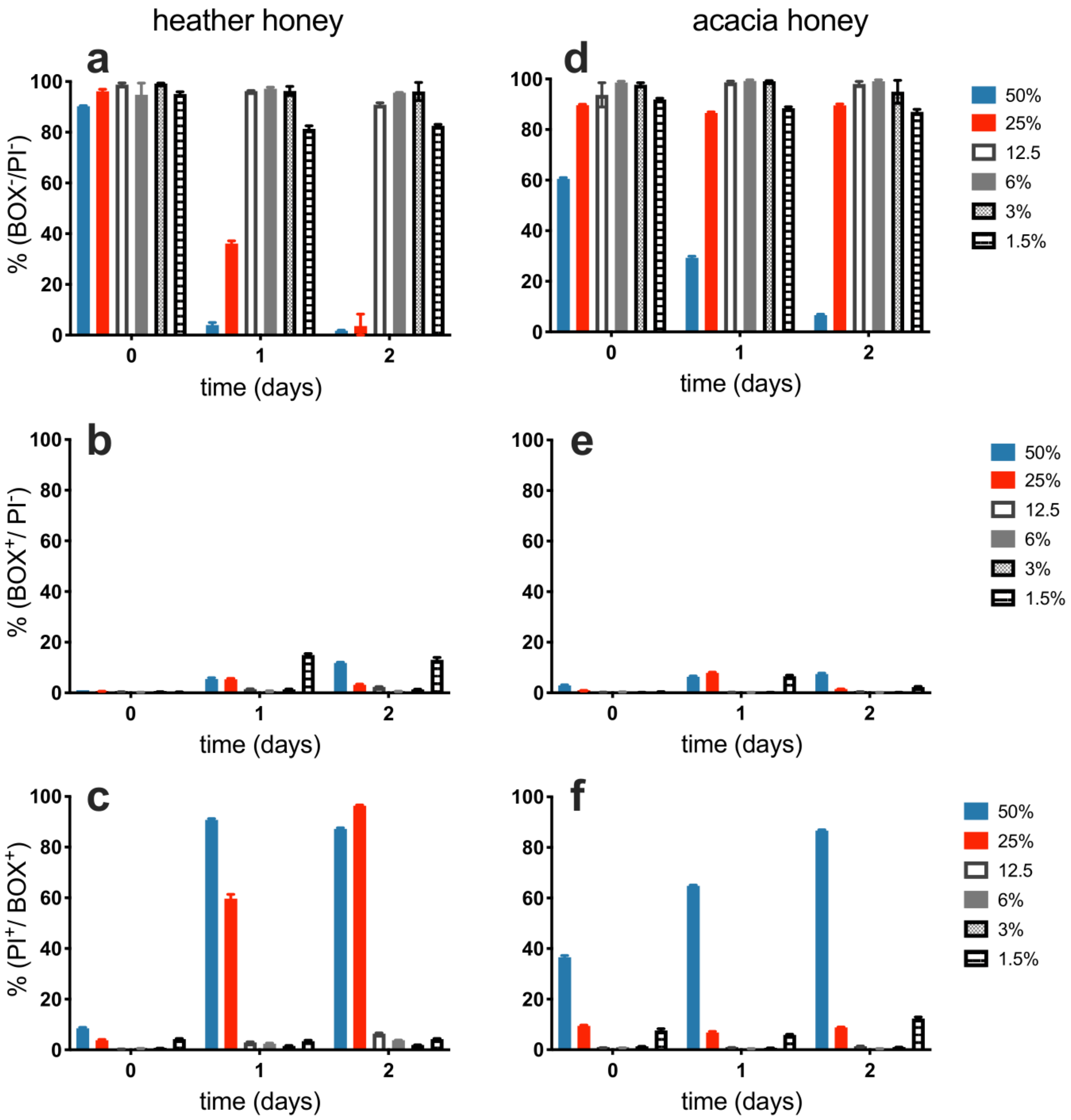

Supplementary Figure S4: FC analysis on physiology of *E.coli* MG1655 exposed to serially diluted (50-1.5%) heather and acacia honey. Double staining (PI/BOX) discriminates three populations; (**a, d**) "healthy"/viable (PI^-/BOX^-), (**b, e**) "injured "(or depolarised) (PI^-/BOX^+), and (c, f) "dead" (or membrane destructed) (PI^+/BOX^+). Error bars represent the mean ± SD. of three (*n*=3) biological replicates.

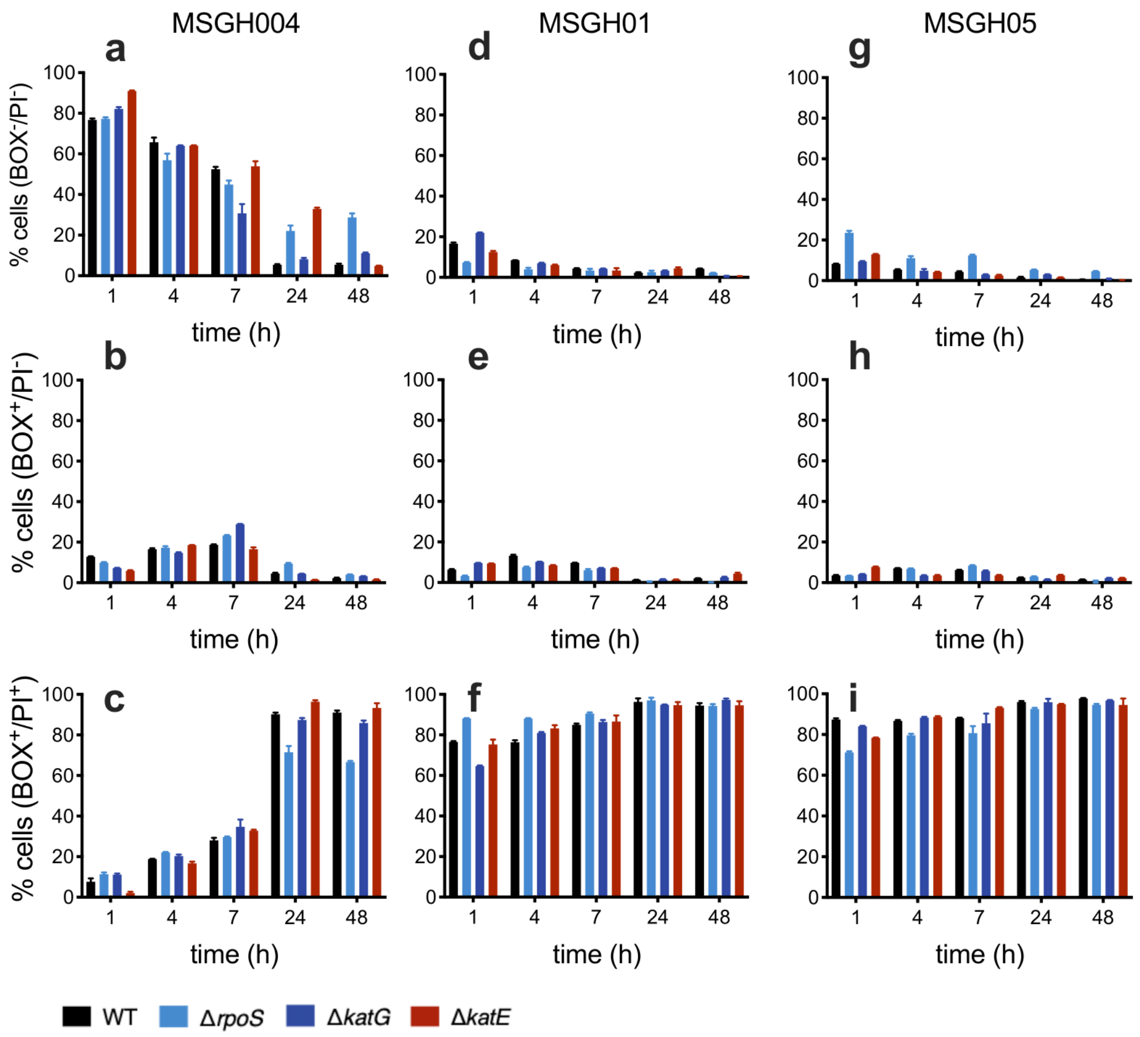

Supplementary Figure S5. FC analysis on physiology of *E. coli* K-12 WT, Δ*rpoS* and catalase depleted (Δ*katG,* Δ*katE*) mutants post exposure to model honey of increasing H_2O_2 concentration. Double staining (PI and BOX) discriminates three populations; (**a, d, g**) "healthy"/viable (PI^-/BOX^-), (**b, e, h**) "injured" (or depolarised) (PI^-/BOX^+), and (**c, f, i**) "dead" (or membrane destructed) (PI^+/BOX^+). The composition of the three model honeys, MSGH00, MSGH01 and MSGH05 is given on Table 1. Error bars represent the mean ± SD. of three (*n*=3) biological replicates.

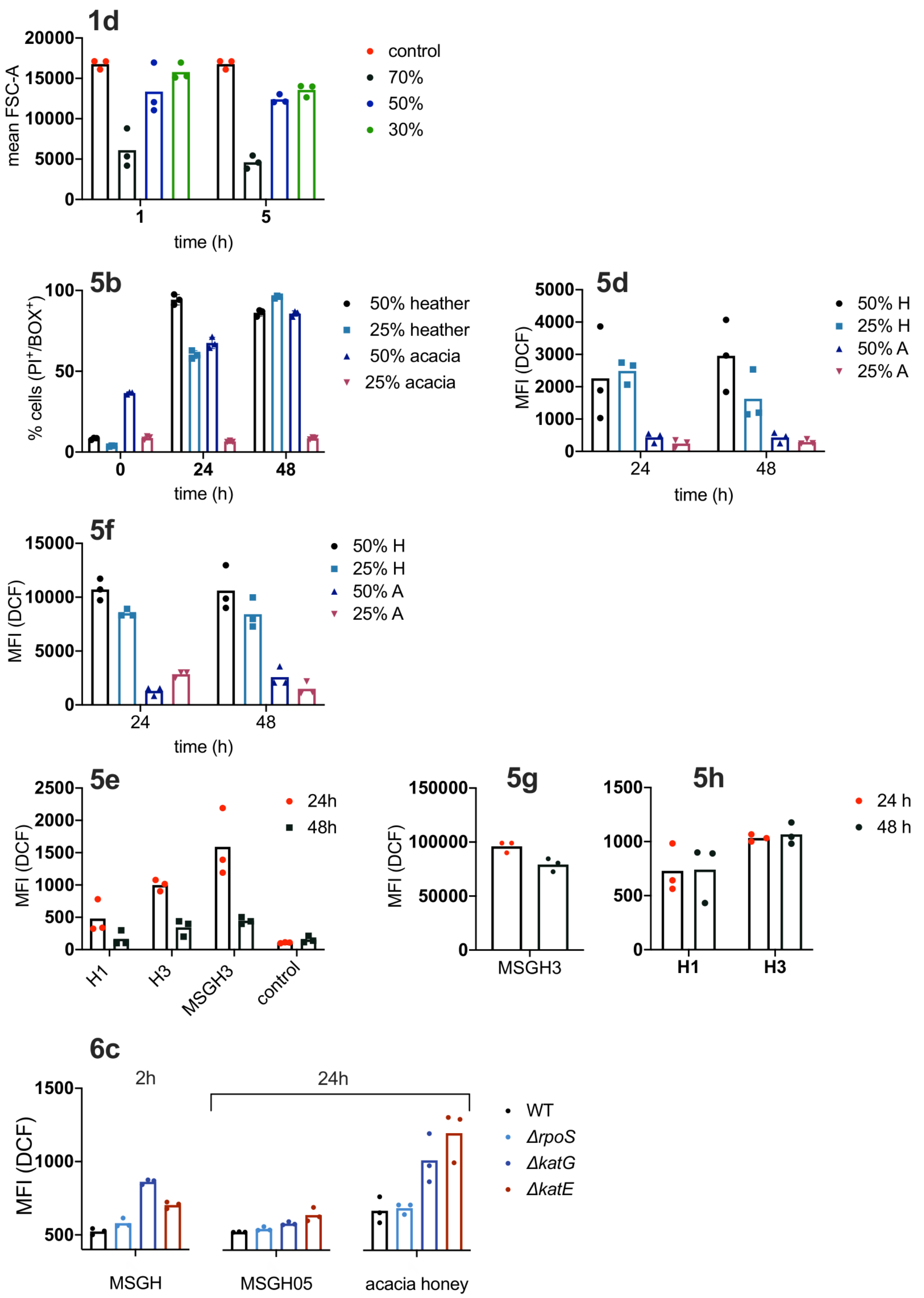

Supplementary Figure S6. Original data. These graphs show the variation of three biological replicates executed in each experiment. Each graph is annotated as such is referred within the main manuscript.

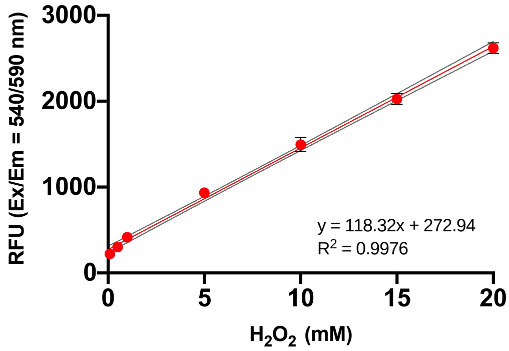

Supplementary Figure S7. Fluorometric standard curve for a range of H_2O_2 concentration between 0-20 mM. Standard H_2O_2 solutions (0.1, 0.5, 1, 5, 10, 15 and 20 mM) were prepared and mixed with peroxidase substrate. The reaction generates a red fluorescent product (Ex=540 nm/ Em=590 nm) which was analysed by the fluorescent microplate reader (CLARIOstar; BMG Labtech, US).
